# Low-dose X-ray therapy for COVID-19: lessons from the past

**DOI:** 10.1259/bjr.20200581

**Published:** 2020-11-30

**Authors:** Awais Nasir, Deepsha Agrawal, Jayashree Pathak, Iakovos Theodoulou

**Affiliations:** 1 Cambridge University Hospitals, Cambridge, UK; 2 Oxford University Hospitals, Oxford, UK; 3 Ysbyty Ystrad Fawr Hospital, Ystrad Mynach, UK; 4 School of Medicine, King’s College London, London, UK

## Abstract

Coronavirus disease 19 (Covid-19) poses a huge threat to health systems and economies worldwide. So far, there has been no proven effective treatment for SARS-CoV-2 infection. Various potential therapies, viz., immunomodulatory agents, antiviral therapy, and plasma transfusion, are undergoing clinical trials. An intensive search of the medical corpora revealed that low dose X-ray radiation therapy has been used in the past to treat interstitial pneumonia. In this article we explore a historical background of low-dose X-rays for the treatment of pneumonia and how it could be a promising therapy in treating patients with COVID-19.

## Introduction

The highly contagious SARS-CoV-2 virus has afflicted the entire world and as of 31 August 2020 has resulted in 25,251,334 cases of COVID-19 including 846,841 deaths.

Comprehensive data on any effective drug or therapy for SARS-CoV-2 infection are still lacking. Several large-scale, multicenter trials are underway to study several proposed therapeutic options, including the WHO Solidarity Trial, and the UK Recovery Trial. The Recovery Trial is currently testing some of these suggested treatments, low-dose Dexamethasone, Azithromycin, Tocilizumab, and convalescent plasma.

At this stage, a revisit to the medical corpora and studying traditional therapies may yield some answers. There has been no explicit study on the mechanism of action of X-rays on resolution of the pneumonia. However, research points towards anti inflammatory effects of low doses of ionizing radiation as a principal mode of action. PaO2/FiO2 ratio is the ratio of arterial oxygen partial pressure (PaO2 in mmHg) to fractional inspired oxygen. Low-dose lung irradiation in COVID-19 interstitial pneumonia may work as an adjunctive treatment by improving the PaO2/FiO2 ratio, thereby reducing the number of days of ventilator requirement.

The SARS-CoV-2 primarily infects primarily bronchial and alveolar epithelial cells. High viral replication in these cells induces cytopathic effects followed by activation of an inflammatory cascade leading to an accelerated inflammatory state. Irradiation significantly decreases the proliferative response of lymphocytes. It is postulated that the anti-inflammatory effect of low-dose X-rays is mediated by the modulation of oxidative burst and suppression of pro-inflammatory cytokines and induction of regulatory T cells.^1^

### Lessons from the past

The use of low doses of X-rays to treat patients with pneumonia goes back in time to the first half of the 20th century. Historically, the first ever report was by Musser and Edsall^2^ in 1905 at the University of Pennsylvania, who assessed five cases of bacterial pyogenic pneumonia. They hypothesized that X-rays augmented metabolic processes in cells and accelerated resolution of pneumonia. Metabolism was estimated by measuring differentials in urinary excretion of nitrogen, chloride, uric acid, and phosphorus. They observed and reported a favorable influence of low dose X-rays in four cases of delayed resolution of lobar pneumonia.

The valuable observations of Musser et al led to another study nearly 10 years later. In 1916, Quimby and Quimby^3^ reported successful treatment of 12 cases of unresolved pneumonia using X-rays. In 1924, Heidenhain and Fried reported a study in which 243 acute and subacute pyrogenic infections were successfully treated with X-rays.^4^ They observed that not only did the X-ray treatment was curative but also it was effective when other treatments did not render any benefit such as in cases of chronic bronchopneumonia. Furthermore, the disease resolution was achieved with a single dose of X-ray therapy.

In the fall of 1923, a diagnostic lung X-ray of a child with pneumonia seemed to be a factor in the resolution of pneumonia. The ill child showed signs of resolution 24 h after the mild dose of X-ray. This was observed by Krost who subsequently studied the capacity of X-ray treatment to accelerate recovery of unresolved pneumonia in children.^5^ He reported that 11 out of 12 patients displayed notable improvement following the treatment.

In January 1933, Eugene Powell obtained permission from his physician-in-charge to try low-dose X-ray therapy in patients of lobar pneumonia. Based on the premise of some relation between the destruction of the infiltrating leukocytes and the resolution of the inflammatory condition he employed the therapy on a patient who was ill with lobar pneumonia. Quoting from Powell: “Unable to find any references in the literature to guide me in dosage, I used a technique which had proved valuable in the treatment of carbuncles. However, I increased the filtration and skin-target distance, so as to irradiate more homogeneously the large mass of tissue that is involved in a consolidated pulmonary lobe. Within a few hours after the treatment the patient was relieved of much of his distress, and within 24 h his temperature dropped by crisis. He then pursued an uneventful and complete convalescence.” He then used this technique on 134 patients and demonstrated a notable reduction in mortality.^6^

Interestingly, Powell had intended to employ X-ray therapy in alternative cases of pneumonia establishing a test and control group. However, the reduction in mortality rate and distress was so remarkable that it was deemed unethical to deny such beneficial treatment.

Following this Oppenheimer,^7^ in 1943, studied the effect of X-ray therapy on interstitial pneumonia failing to respond to medical treatment. The study included pediatric patients from 2 days old to 13 years. The diagnosis was based upon the presence of fever, cough, and dyspnea, associated with infiltration along and around the bronchi and bronchioles radiologically, with absence of lobar and bronchopneumonia consolidations, and failure of the signs and symptoms to respond to sulfonamide medication.

The dosage was determined by the age of the patient and duration of the disease. As soon as the diagnosis was established, the patient was given a single dose of X-ray treatment with doses between 0.1754 and 0.7893 Gy. With his therapy, he treated 56 patients that had failed to respond to various standard medical treatments, including antibiotics therapy.

Although the results of this therapy were very promising, the advent and wide scale production of novel antibiotics in the period between the 1950s and 1970s, offered a less costly treatment for pneumonia. Hence, studies on low dose X-ray therapy for pneumonia were discontinued, just like the serum therapy.

As a consequence, it never picked up and further exploration into this subject gradually died. While these studies present substantial findings regarding the role of X-rays in the treatment of pneumonia, there is a lacuna in robust data on long-term risks of such therapy. Within the context of the current COVID-19 pandemic and an ongoing global health crisis, this therapy now merits contemporary research.

Currently, many such low dose radiation therapies for COVID-19 patients, using upto 1 Gy of a single dose, are underway (see, *eg* Clinicaltrials.gov*.*^8^).

Several animal model studies have also been conducted and reported. There are two published reports on utilizing low dose radiotherapy for viral pneumonia in preclinical models.^9,10^ These have demonstrated some measure of support for the hypothesis that X-ray treatment could reduce the effects of viral pneumonia.

The dose, mode of delivery and radiotherapy schedule might be important determinants in the anti-inflammatory effects of low dose radiation. Moreover, there is mounting evidence that local radiation recruits biological effectors outside the treatment field that results in systemic anti-inflammatory effects, a phenomenon known as systemic immune-related response. Secondary malignant neoplasms (SMNs) are among the most serious consequences of tissue irradiation. The risk of SMNs increases with increasing radiation field size. Although not studied for low doses irradiation approach at low linear energy transfer (LET) for pneumonia treatment, for patients with Hodgkin’s lymphoma the relative risk doubled from mantle radiation to subtotal lymph node radiation.

It is notable that the average duration of illness was longer in the animals that received radiation 48 h after symptom onset and did not differ significantly from the control group. This indicates that radiation therapy for viral pneumonia may need to be initiated early. However, these studies have important limitations like reproducibility and lack of double-blinding.

In summary, the available preclinical and clinical data suggest further research into this therapy.
